# Evaluation of the subcapsular technique for primary closure castration in donkeys (*Equus asinus*)

**DOI:** 10.1038/s41598-021-93585-y

**Published:** 2021-07-07

**Authors:** Ahmed Ibrahim, Usama T. Mahmoud, Magda M. Ali, Sohair M. M. Ragab

**Affiliations:** 1 Veterinary Teaching Hospital, Faculty of Veterinary Medicine, Assiut University, Assiut, 71526 Egypt; 2 Department of Animal and Poultry Behavior and Management, Faculty of Veterinary Medicine, Assiut University, Assiut, 71526 Egypt; 3 Department of Surgery, Anesthesiology and Radiology, Faculty of Veterinary Medicine, Assiut University, Assiut, 71526 Egypt; 4 Department of Zoology, Faculty of Science, Assiut Uuniversity, Assiut, 71526 Egypt

**Keywords:** Biological techniques, Medical research

## Abstract

This study described the subcapsular technique for primary closure castration in donkeys with special regard to its efficiency and welfare impacts. The study was conducted on twelve adult male donkeys, allocated randomly into two groups; subcapsular castration (SC) and open castration (OC) groups, whether the donkeys were subjected to surgical castration either by subcapsular or open castration techniques, respectively. Testosterone, cortisol, lactate, glucose, total cholesterol (TC), high density lipoprotein cholesterol (HDL-C), triglyceride (TG), and nitric oxide (NO) were measured before and after castration. Pain-associated behavioral activities were recorded post-castration. The SC was successfully performed in donkeys through a single paramedian scrotal incision. The SC was efficient as OC in reducing testosterone levels. The pain score decreased in the SC compared to the OC over time. The SC was an efficient and reliable technique for primary closure castration in donkeys with minimal postoperative complications and care and good cosmetic, physiological, and behavioral outcomes. It can be an alternative to other castration techniques in equines.

## Introduction

Castration is a surgical practice frequently performed in male equids for the elimination of undesirable sexual behavior or surgical management of testicular tumors and trauma, orchitis, inguinal hernia, hydrocele, varicocele, and spermatic cord torsion^1–3^. Several studies have described different procedures for surgical castration of equines, including; the open, modified open, closed, semi-closed (either via the scrotal or inguinal approach), section-ligation-release (SLR), and laparoscopic techniques in standing, lateral, or dorsal positions^2–9^.

However, surgical castration is associated with different postoperative complications in equines, involving hemorrhage, infection (incisional infection and scirrhous cord), eventration, excessive edema, and peritonitis^1,3,5,10,11^, which occasionally require additional surgical interference or intensive medical management^12,13^.

Although donkeys are known to mask and hide symptoms of pain^14^, castration of male donkey has been shown to elicit physiological stress and pain-associated behavioral activities^14–16^. Attempts to alleviate the undesirable effects of castration in donkeys have been done with varying degrees of success^15^.

Subcapsular castration is a technique frequently used in human patients with prostate cancer to avoid the psychological consequences of the empty scrotum that result from total orchiectomy. It is accomplished through a small scrotal incision^17–21^.

Currently, no literature has addressed the subcapsular castration technique in equines. We hypothesized that excision of testicular parenchyma bilaterally through a primary closed single scrotal incision in the subcapsular castration can be efficient and minimize postoperative complications and care in castrated donkeys. Therefore, the main aim of this study was to describe the subcapsular technique for primary closure castration in donkeys (*Equus asinus*) with special regard to its efficiency and welfare impacts.

## Materials and methods

### Ethical approval

The National Ethical Committee of The Faculty of Veterinary Medicine, Assiut University, Assiut, Egypt, has approved all the procedures in this study in accordance with the Egyptian bylaws and OIE animal welfare standards for animal care and use in research and education. This study was carried out in compliance with the ARRIVE guidelines.

### Animals

This study was conducted on twelve clinically healthy adult male donkeys (*Equus asinus*) with normally descended testicles on physical examination, aged 6–8 years and weighed 150–175 kg. They were obtained from the Experimental Animal House, Veterinary Teaching Hospital, Faculty of Veterinary Medicine, Assiut, Egypt, and housed in standard stables with ad libitum access to water. Donkeys were fed concentrate ration twice daily and were randomly allocated into two groups; subcapsular castration (SC) and open castration (OC) groups (each of six donkeys), whether subjected to surgical castration either by subcapsular castration or open castration techniques, respectively. All animals received prophylactic tetanus antitoxin (3000 IU, subcutaneously) before surgery.

### Surgical procedures

#### Anesthetic protocol

Feed and water were withheld for 24 and 2 h, respectively, before surgery. Donkeys were sedated with intravenous 1.1 mg/kg xylazine HCl 2% (Xyla-Ject, ADWIA Co., Egypt). Anesthesia was induced with 2.2 mg/kg ketamine HCl 5% (Ketamine, Sigma-tec Pharmaceutical Industries, Egypt) combined with 0.01 mg/kg diazepam 0.5% (Neuril, Memphis, Egypt) given intravenously. Anesthesia was then maintained with triple drip mixture (12.5 g guaifenesin [Guaifenesin, powder, Unidrug Innovative Pharma Technologies Limited, India], 500 mg ketamine HCl 5%, and 150 mg xylazine HCl 2%, combined in 500 ml NaCl 0.9%, given at 1 ml/kg/h)^22,23^. Ten ml lidocaine HCl 2% (Dibucaine, Sigma-Tec Pharmaceutical Industry Co., Egypt) was infiltrated into each testis and spermatic cord and 5 ml subcutaneously over the anticipated site of incision. The anesthetized donkey was placed in the left lateral recumbency with the uppermost hind limb tied cranially. The surgical area was aseptically prepared for surgery and draped except for the scrotum. The lower testis was first operated in both techniques. Surgical time (min) was measured (from skin incision to skin closure).

#### Subcapsular castration technique

**Figure 1:**
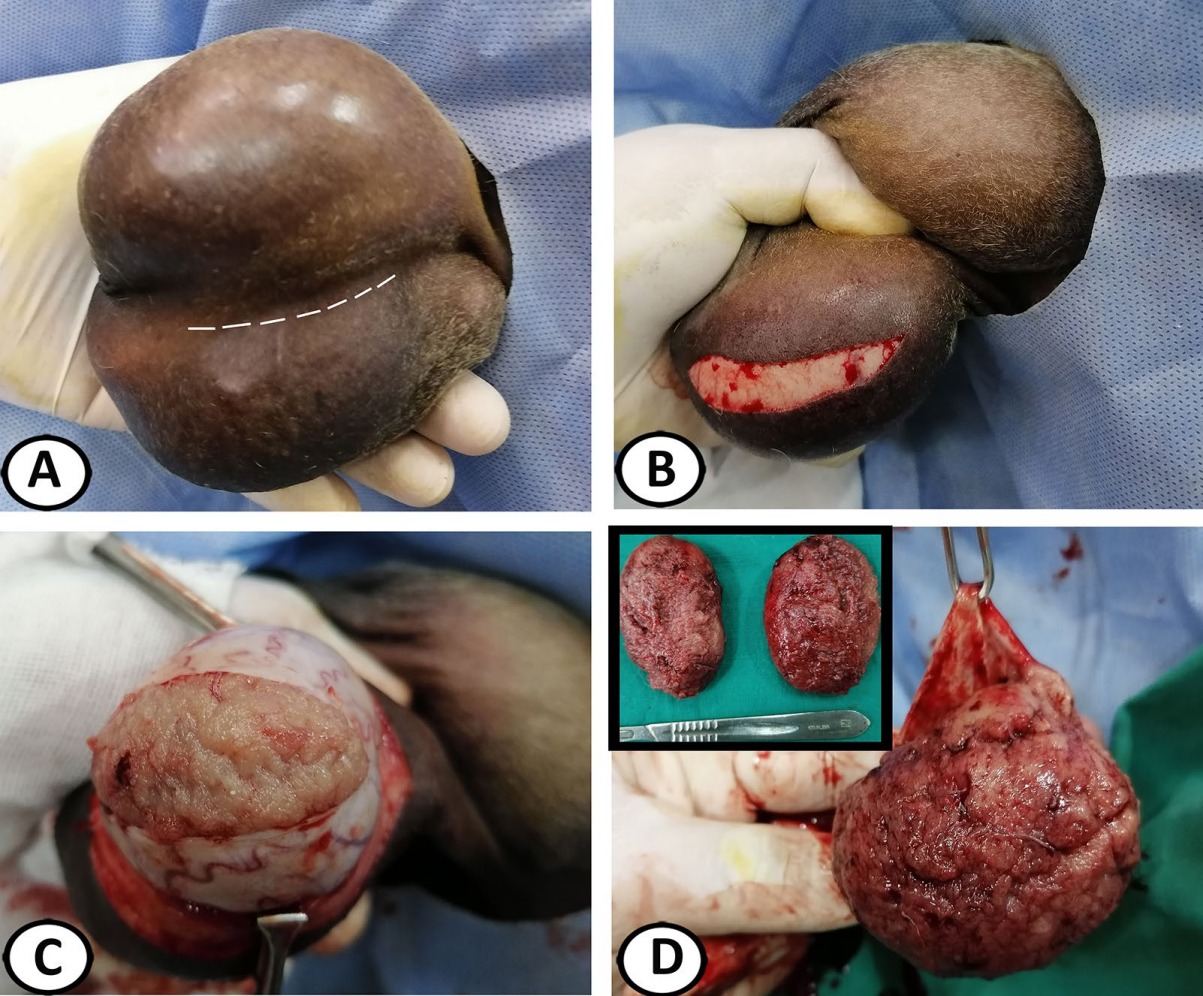
Subcapsular castration (SC) in donkeys. (**A**) Anticipated site of the scrotal incision. (**B**) Incision of the scrotal skin. (**C**) Incision of the dartos fascia, external and internal spermatic fasciae, parietal and visceral vaginal tunics, and tunica albuginea. (**D**) Surgical dissection of the testicular parenchyma. Note, the excised testicular parenchyma (inserted figure).

**Figure 2:**
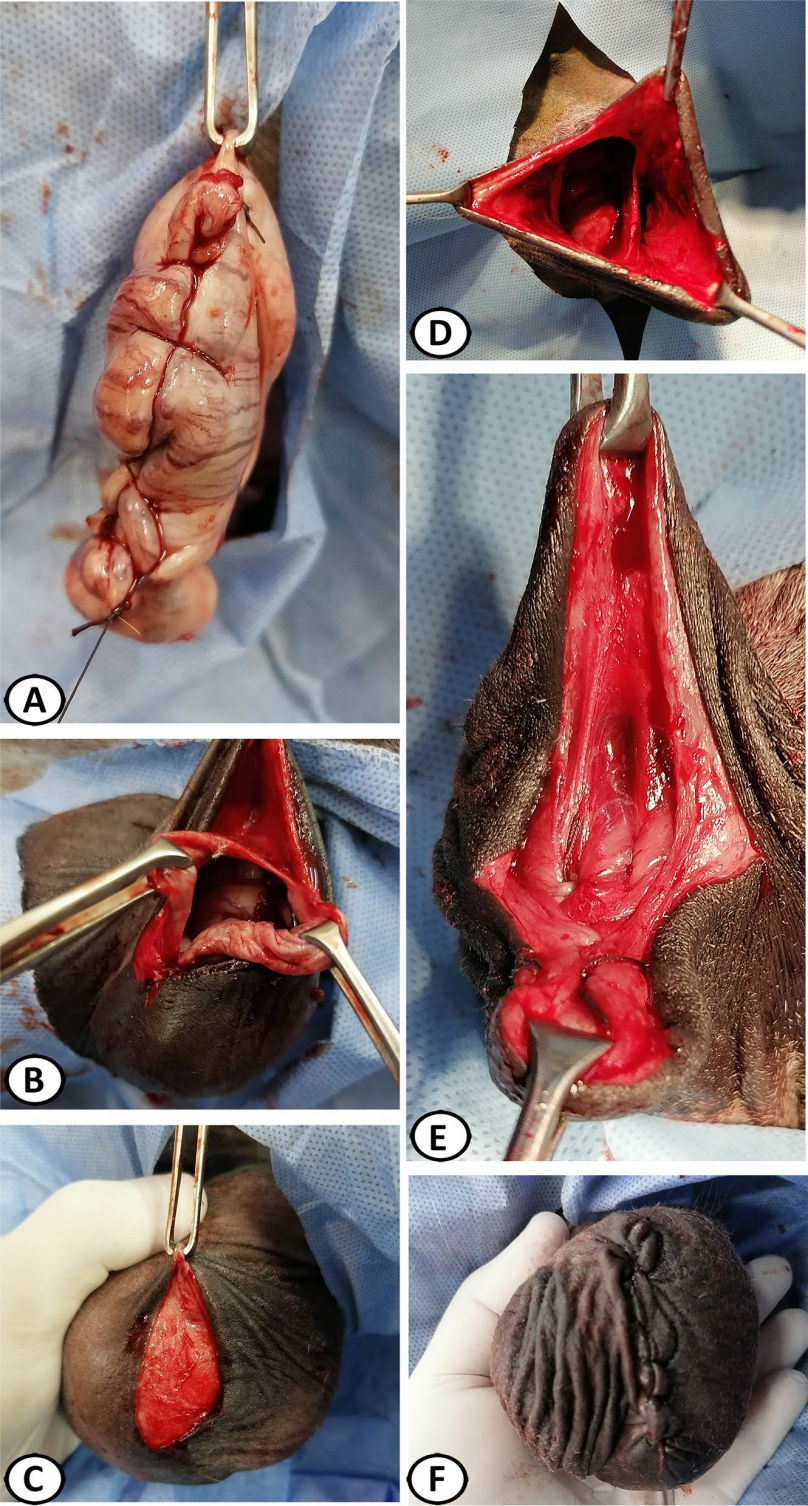
Subcapsular castration (SC) in donkeys. (**A**) Closing of the albuginea and visceral vaginal tunic. (**B**) The operated testicle returned into its pouch. (**C**) Access to the second testicle by cutting the median sagittal septum, entering the vaginal cavity of the first scrotal pouch while comprising it. (**D**) The second operated testicle returned into its pouch. (**E**) Closing of the median sagittal septum and the opened scrotal pouch, formed by internal spermatic fascia and parietal vaginal tunic. (**F**) Closing of the scrotal skin.

A single craniocaudal incision (6–7 cm) was created over the lower testicle, 1 cm apart from and parallel to the median raphe through the scrotal skin, dartos fascia, external and internal spermatic fasciae, parietal and visceral vaginal tunics, and tunica albuginea, while compressing the testicle into the scrotum (Fig. 1A–C). The testicular parenchyma was bluntly dissected from the tunica albuginea (Fig. 1D). Bleeding points in the tunica albuginea were cauterized by the diathermy following surgical excision of the testicular parenchyma. The tunica albuginea and visceral vaginal tunic were closed with 3–0 USP polyglactin 910 (EGYSORP, TAISIER-MED, Egypt) in a Connell suture pattern and returned into their pouch (Fig. 2A,B). Access to the second testicle was done by cutting the median sagittal septum, entering the vaginal cavity of the first scrotal pouch while comprising it (Fig. 2C), treated with the same procedure, and returned into its pouch (Fig. 2D). The septum and opened scrotal pouch, formed by internal spermatic fascia and parietal vaginal tunic, were independently closed with 3–0 USP polyglactin 910 in a simple continuous pattern (Fig. 2E). The skin was closed with 0 USP polyglactin 910 in a simple continuous pattern (Fig. 2F)^20^.

#### Open castration technique

A craniocaudal incision over the lower testicle, 1 cm apart from and parallel to the median raphe was created through the scrotal skin, dartos fascia, external and internal spermatic fasciae, and parietal vaginal tunic, while compressing the testicle into the scrotum. The testicle was exteriorized through the incision after disruption of the ligament of the tail of the epididymis and mesofuniculus. An opening was created in the mesorchium to introduce a circumferential ligature using 2 USP polyglactin 910 around the spermatic vessels. The spermatic vasculature was then crushed independent of the ductus deferens and mesorchium. The emasculator was applied distal to the ligature on the cord for two minutes before severing it. The cord bud was inspected for hemorrhage. Any exposed tunic was crushed and removed. The procedure was then repeated on the second testicle. Wounds were left open to heal by the second intention^2,12^.

#### Postoperative care and evaluation

Donkeys were kept in the lateral recumbency after surgery in a well-padded recovery stall and monitored until they were standing. Wounds were monitored carefully 2–3 times daily postoperatively for signs of bleeding, excessive swelling, or infection. Scrotal edema was scored on a scale of 0–3 (0 = no edema, 1 = mild scrotal edema did not extend to the prepuce, 2 = moderate scrotal edema slightly extended to the prepuce, and 3 = severely diffused scrotal and preputial edema). Donkeys received intravenous 1.1 mg/kg flunixin meglumine 5% (Flunixin, Norbrook, Ireland) twice daily and intramuscular 10 mg/kg amoxicillin/gentamycin combination (Gentamox, Amoxicillin 150 mg/ml, Gentamycin 40 mg/ml, Hibra, Spain) once daily on the day of surgery and for 5 successive days postoperatively. Donkeys of both groups had 10–15 min walking in hand twice a day for 5 successive days post-surgery.

### Biochemical measurements

#### Collection of blood samples

Blood samples were collected from the jugular vein under complete aseptic conditions at seven-time points; before (C) and at the end of the surgery (E), then at 12 h, 2, 7, 15, and 30 days post-surgery. Serum was isolated after centrifugation at 3000 rpm for 10 min and stored at − 20 °C until analysis for determination of biochemical parameters.

#### Biochemical parameters

Testosterone and cortisol levels were measured by Cobas e411 (Hitachi High-Technologies Corporation, Tokyo, Japan). Lactate, glucose, total cholesterol (TC), high density lipoprotein cholesterol (HDL-C), and triglyceride (TG) levels were measured according to the manufacturer’s instructions using commercially available kits (Egyptian Company for Biotechnology, Egypt). Enzymatic colorimetric method using lactate oxidase and 4-aminoantipyrine was used for estimation of lactate level. Glucose level was determined after enzymatic oxidation in the presence of glucose oxidase. The formed hydrogen peroxide reacts under the catalysis of peroxidase with phenol and 4-aminoantipyrine to form a red-violet quinoneimine dye as an indicator. TC was estimated using cholesterol oxidase/peroxidase aminophena-zone reagent. HDL-C was assessed by the precipitation method. Briefly, low-density lipoproteins and very low-density lipoproteins in the sample were precipitated with phosphotungstate and magnesium ions. After centrifugation, the cholesterol concentration in the HDL fraction, which remained in the supernatant was determined. Glycerine phosphate oxidase peroxidase colorimetric method was used for the measurement of TG. Nitric oxide (NO) level was colorimetrically measured as nitrite concentration according to Ding et al.^24^. In this method, phosphoric acid is quantitatively converted to a diazonium salt by reaction with nitrite in acid solution. The diazonium salt is then coupled to naphthylene diamine dihydrochloride forming an azo dye that can be spectrophotometrically quantitated based on its absorbance at 550 nm.

#### Behavioral evaluation

The ethogram used in this study to evaluate the post‐operative pain behavior associated with surgical castration in donkeys was developed from the previous study by Oliveira et al.^16^ (Table 1). Continuous behavioral observations were conducted for 10 min for each donkey twice daily (8:00–10:00 AM and 16:00–18:00 PM) (time of providing the feed); for six sessions on three consecutive days (so each animal was observed for total 1 h/week). Behaviors were recorded directly onto a check sheet by a single observer. A focal sampling of pre-defined event behaviors was recorded. Standing, lying, feeding, and drinking times were recorded using a stopwatch throughout the 10-min period. Walking, grooming, tail wagging, exposing penis, testicles suckling, and pelvic limb lifting were recorded as frequency/h. Each donkey was identified by a number using coloring livestock spray.

**Table 1:** Ethogram of donkeys subjected to subcapsular (SC) and open castration (OC) techniques and their description (Oliveira et al., 2020).

Behaviors	Description of behaviors
Feeding	The animal is feeding
Water drinking	The animal drinks water
Tail wagging	The animal shakes its tail Tail moves swiftly from its base in a side-to-side flicking manner around the hindquarters^51^
Pelvic limb lifting	The animal lifts one of the pelvic limbs
Exposing penis	The animal exposes its penis without urinating
Grooming	The animal rubs its head on the food trough The animal rubs its head on the wall of the stall The animal scratches its head with one of its pelvic limbs The animal bends and scratches any part of its chest or abdomen with its head
Testicle suckling	The animal lowers its head and directs it to the testicular area and nibble the testicles by its lips
Walking	The animal walks freely in the stall
Standing	Standing. This posture was defined as the sum of standing normally and standing abnormally. Standing normally was further divided into standing passively with no obvious abnormality and standing actively feeding, drinking, or grooming. Standing abnormal was further classified as standing stationary with no movement of legs or body, sometimes with a hunched back or trembling, and standing actively with persistent kicking, foot stamping, or lifting of pelvic legs, tail swishing, or head turned backwards to examine the hind quarters
Lying	This posture was defined as the sum of lying normally and lying abnormally. Lying normally was further classified into lying actively and passive ventral lying with head up or down. Lying abnormally was the same as passive ventral lying with full or partial extension of pelvic legs or lateral lying

#### Pain score

**Figure 3:**
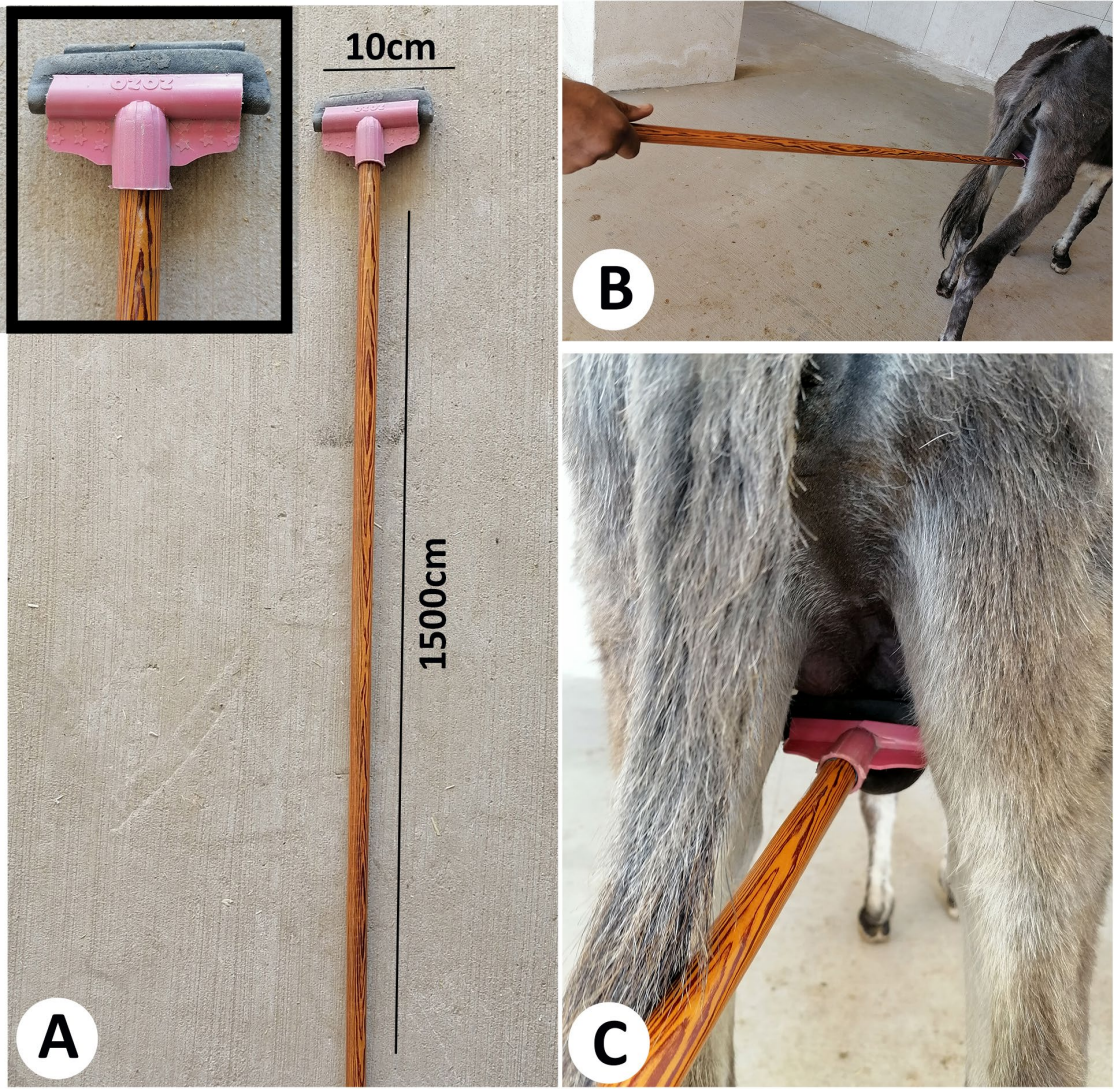
Application of mechanical pressure to the scrotum.

An experienced observer scored pain in donkeys by direct observation on a scale of 0–2 (0 = not present, 1 = moderately present, and 2 = obviously present) according to the donkey grimace scale-scoring system^25^ after the application of a gentle mechanical pressure on the scrotum on days 3, 7, 15 and 30 post-castration (Fig. 3). Scores based on noted signs of discomfort/pain from the observed body language of the face, including; the eye shape (3), orbital tightening (3), ear position (3), and nostril and muzzle tension presences (3), along with their overall body appearance (0 = normal, 1 = abnormal). In addition, the pelvic limb response was scored on a scale of 0–3 (0 = no response, 1 = animal move a step forward to avoid pressure, 2 = lifting the pelvic limb above the ground without kicking, and 3 = kicking). The sum of signs out of the total scores (19) was used to express the pain scale finding. The observer stood about 2 m away from the lateral right side of the donkey to record the animal responses. The test was repeated twice/animal and average score was used for statistical analysis. Average time for each test was 2 ± 0.20 min.

### Statistical analysis

The analysis was performed using GraphPad Prism 5 version 5.01 (GraphPad Software Inc., San Diego, CA, USA). The data were represented as means ± standard error of the mean (SEM). Repeated measures ANOVA was used to determine the effects of each type of castration on the levels of biochemical parameters at each time point relative to the pre-castration one followed by Tukey’s multiple comparison test. Independent-samples t-test was used to compare the effects of each type of castration on the serum biochemical parameters at each time point post-castration. Differences were considered statistically significant for *P* < 0.05. Regarding scrotal edema and pain scores, since data were not normally distributed, a Krustal wallis rank test was used and results were presented as mean rank.

## Results

### Surgical evaluation

The used anesthetic protocol was satisfactory with smooth induction and recovery in all donkeys. There were no recorded deaths between donkeys of the subcapsular castration (SC) or open castration (OC) groups. A single scrotal incision paramedian to the scrotal raphe was satisfactory for approaching both testicles in the SC technique. The SC approach required significantly more time than the OC (43.33 ± 3.33, 9.33 ± 0.66, respectively, *P* < 0.05).

**Figure 4:**
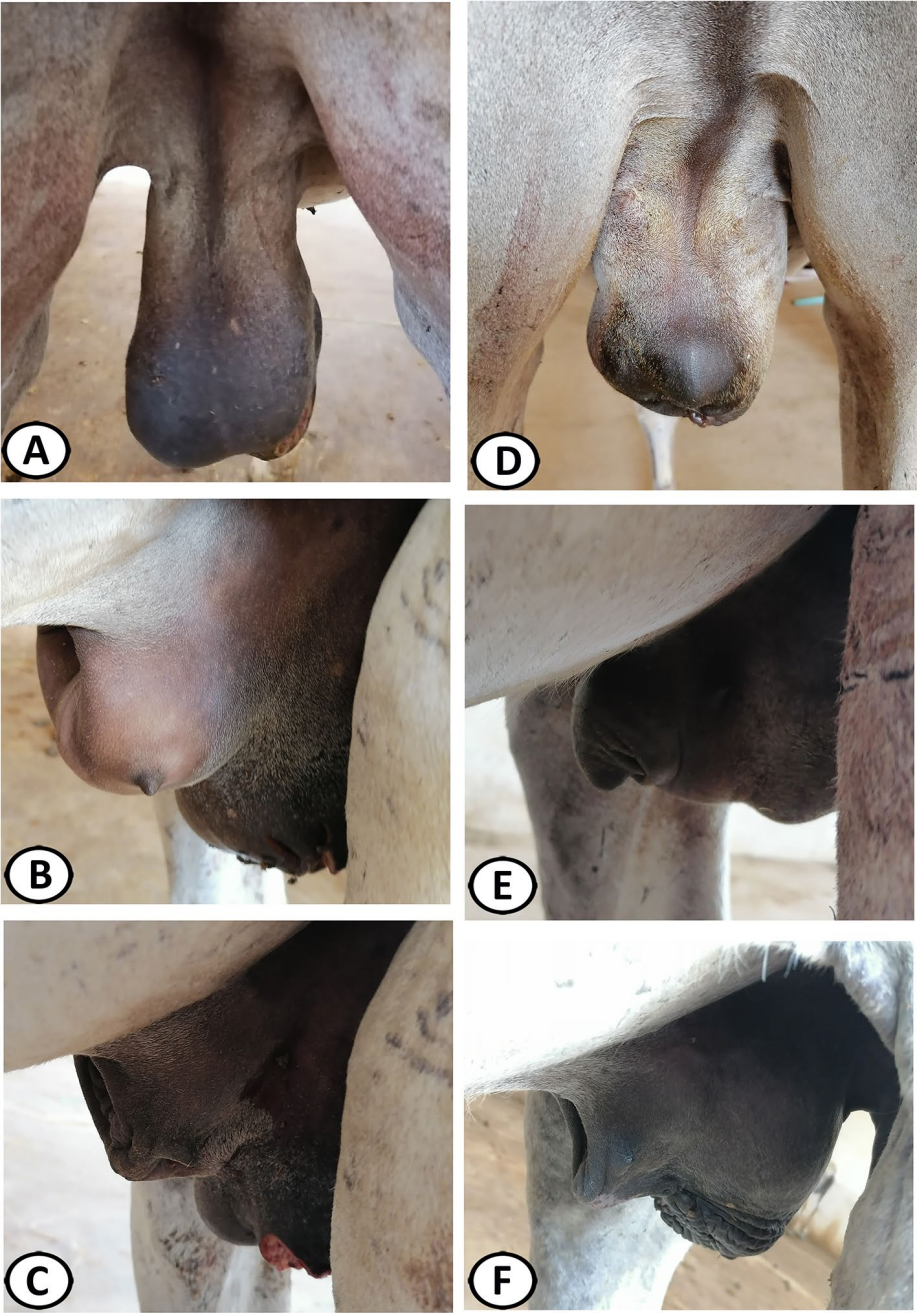
(**A**, **B**) Severely diffused scrotal and preputieal edema in the open castration (OC) group postoperatively. (**D**, **E**) Moderate scrotal edema slightly extended to the prepuce in the subcapsular (SC) group. (**C**, **F**) Resolving of the scrotal and preputial edema in donkeys of the OC (12 days postoperative) and SC (7 days postoperative), respectively.

Donkeys of the OC group exhibited postoperative scrotal and preputial edema more severe than in the SC group (8.67, 4.33, respectively, *P* = 0.02) (Fig. 4A,B and 4D,E, respectively). This edema resolved completely from the genitalia more rapidly in the SC group (6–7 days postoperatively) than in the OC group (10–12 days postoperatively) (Fig. 4C,F). The scrotal wounds healed completely by primary intention (10–12 days postoperatively) without complications (e.g. wound infection or dehiscence) in the donkeys of the SC group.

One donkey in the OC group had a scrotal hemorrhage 12 h postoperatively. There was bilateral scrotal swelling with blood clots and a sluggish but steadily dripping of blood (one drop/second). Further examination of the scrotal wounds revealed that the bleeding originated from blood vessels in the scrotal fascia, which was efficiently managed by direct compression of the bleeding points using a hemostat. The approach was performed while the donkey was in a standing position and under the effect of xylazine HCl 2% (1 mg/kg, intravenously). Furthermore, the co-operative behavior of the animal facilitated surgical interference. Unfortunately, this donkey had a wound infection later that was successfully resolved by maintaining adequate drainage and daily dressing with a diluted povidone-iodine solution until complete recovery. The scrotal wounds of the donkeys in the OC group healed by the second intention and required 20–25 days postoperatively to recover completely. Donkeys of the SC group had a relatively normal scrotal appearance with a fake presence of the right and left testicles on scrotal palpation.

### Physiological findings

**Figure 5:**
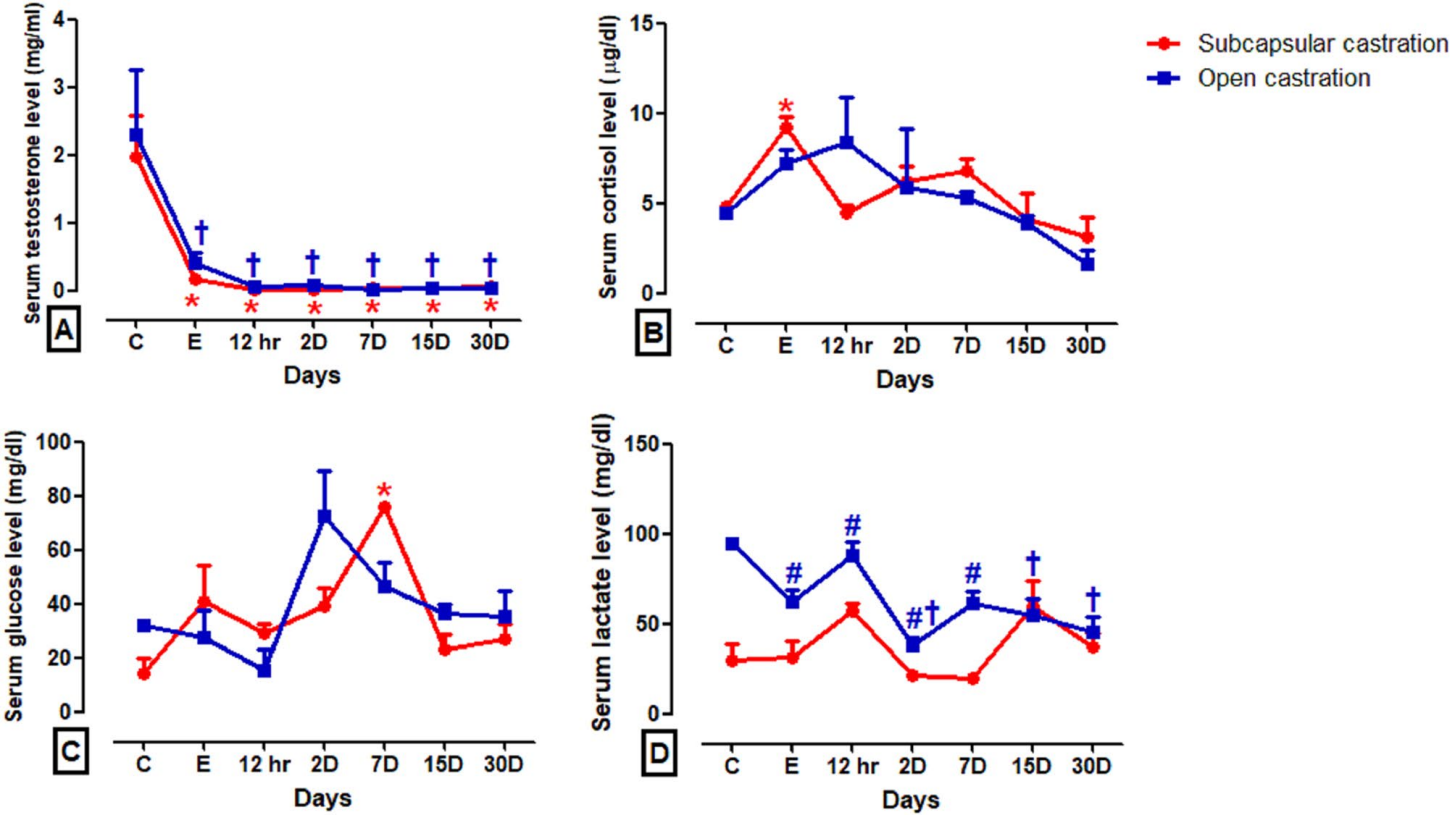
Serum concentrations of testosterone (**A**), cortisol (**B**), glucose (**C**), and lactate (**D**) in the subcapsular castration (SC) and open (OC) castration groups. ^#^Indicates significant difference between different groups, *Indicate significant difference at different time intervals within the SC, ^†^Indicate significant difference at different time intervals within the OC.

The SC technique was as efficient as the OC in significantly reduce (*P* < 0.001) testosterone levels at all-time points post-castration compared to baseline (Fig. 5A). Donkeys subjected to the SC exhibited a significant increase (*P* < 0.01) in cortisol and glucose levels (at the end of the surgery and on day 7 post-castration, respectively) compared to the pre-castration levels (Fig. 5B,C). Lactate levels significantly decreased (*P* < 0.05) at the end of the surgical intervention and persisted for day 7 post-castration (*P* < 0.01) in donkeys subjected to the SC compared with donkeys subjected to the OC. However, lactate levels significantly decreased (*P* < 0.01) on days 2, 15, and 30 post-castration in donkeys of the OC group compared to its baseline (Fig. 5D and S1).

**Figure 6:**
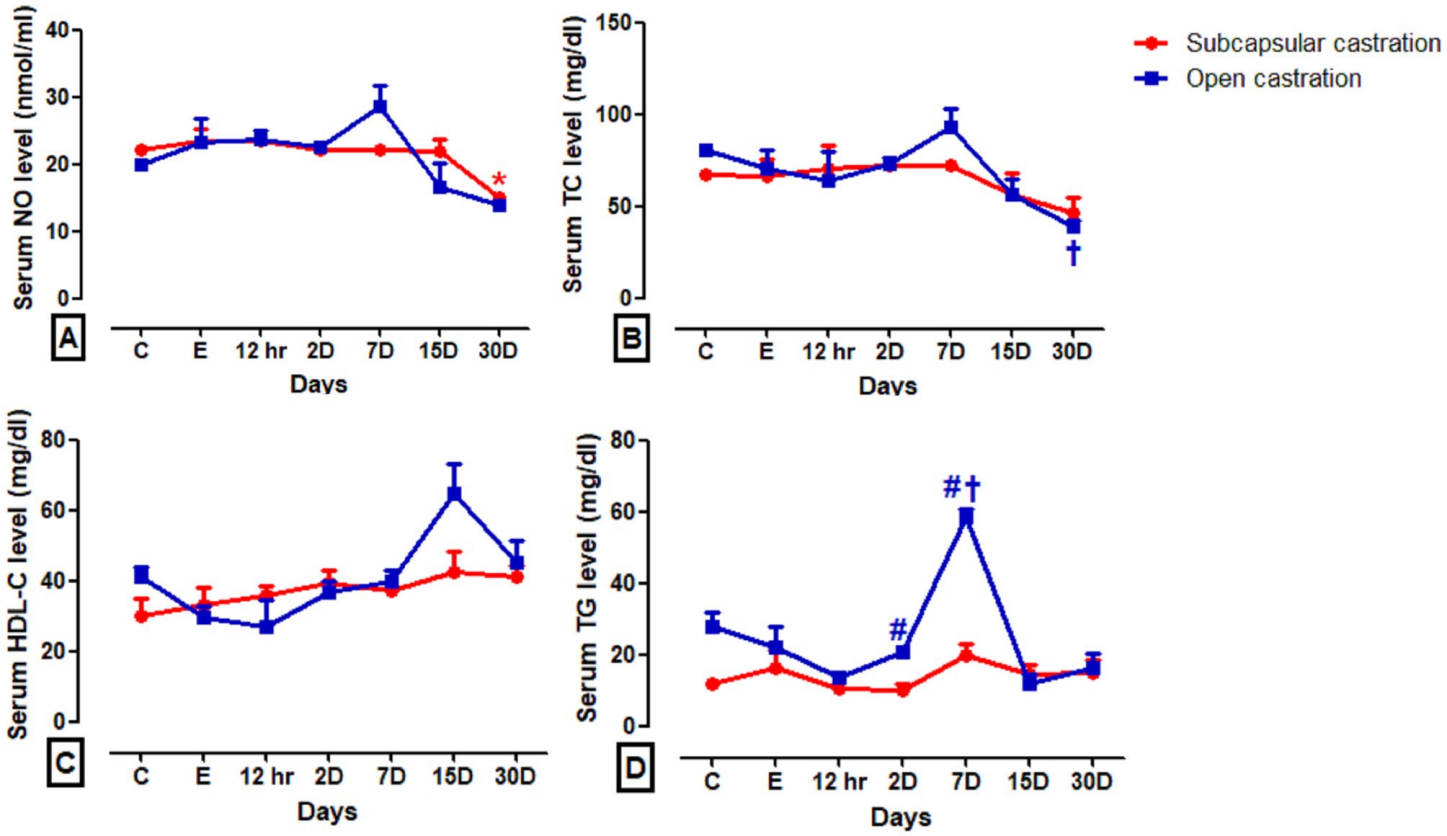
Serum concentrations of NO (**A**), TC (**B**), HDL-C (**C**), and TG (**D**) in the subcapsular (SC) and open (OC) castration groups. ^#^Indicates significant difference between different groups, *Indicate significant difference at different time intervals within the SC, ^†^Indicate significant difference at different time intervals within the OC.

NO and TC levels recorded a significant reduction (*P* < 0.1, *P* < 0.05, respectively) at the last time point in donkeys subjected to the SC and OC, respectively (Fig. 6A,B). Non-significant changes (*P* > 0.05) were recorded regarding HDL-C levels at all post-castration time points relative to the pre-castration level in the same group or between both groups (Fig. 6C). Donkeys of the OC group recorded a significant increase (*P* < 0.001) in TG levels on day 7 post-castration compared to baseline value. However, there was a significant decrease in TG levels on days 2 and 7 post-castration (*P* < 0.05, *P* < 0.001, respectively) in donkeys of the SC compared to donkeys of the OC group (Fig. 6D and S2).

### Behavioral evaluation

**Figure 7:**
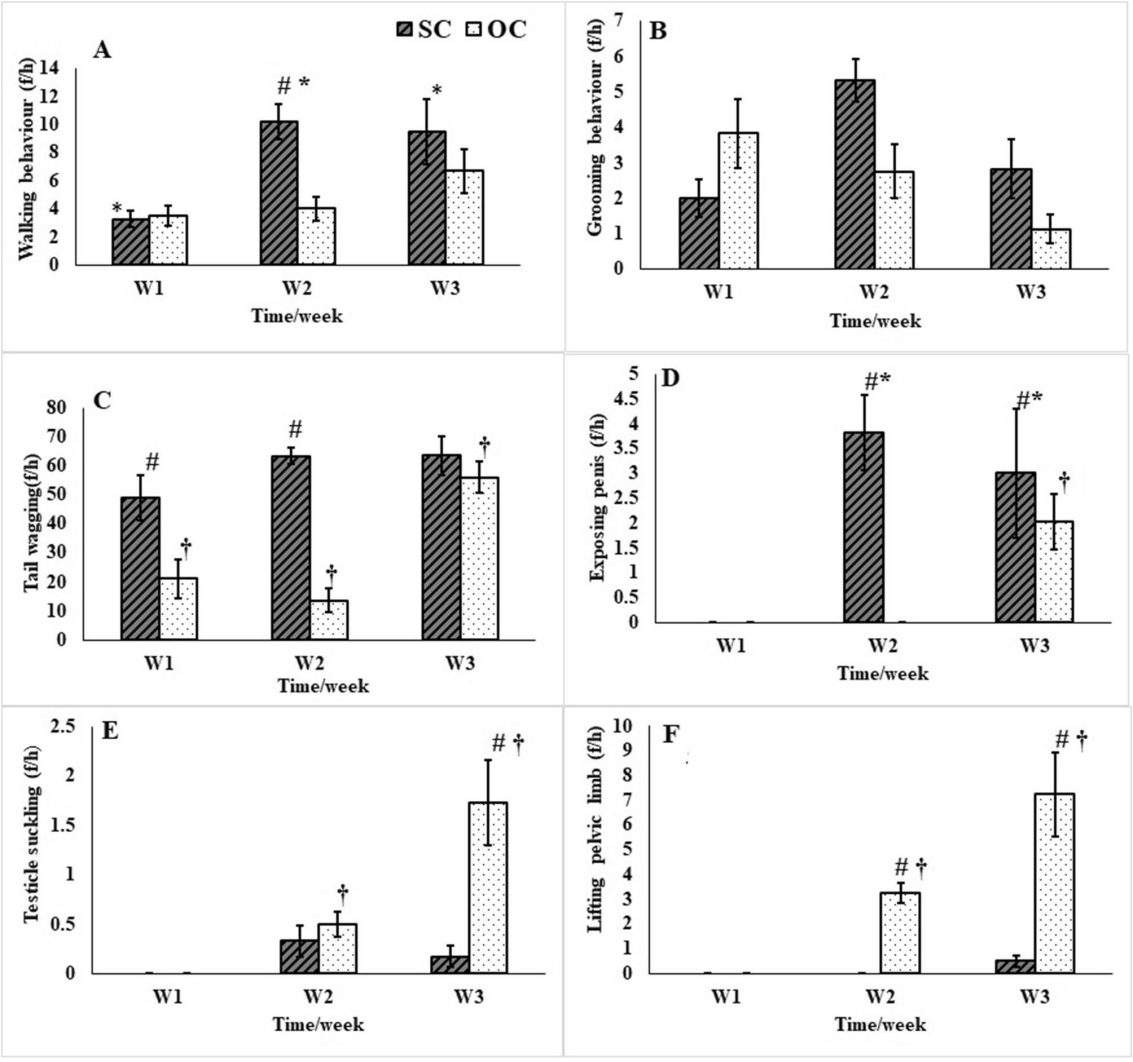
Walking (**A**), grooming (**B**), tail wagging (**C**), exposing penis (**D**), testicles suckling (**E**), lifting pelvic limb (**F**) behavioral activities following subcapsular (SC) and open (OC) castration in donkeys during the 1st, 2nd, and 3rd weeks (W) post-castration. ^#^Indicates significant difference between different groups, *Indicate significant difference at different time intervals within the SC, ^†^Indicate significant difference at different time intervals within the OC.

**Figure 8:**
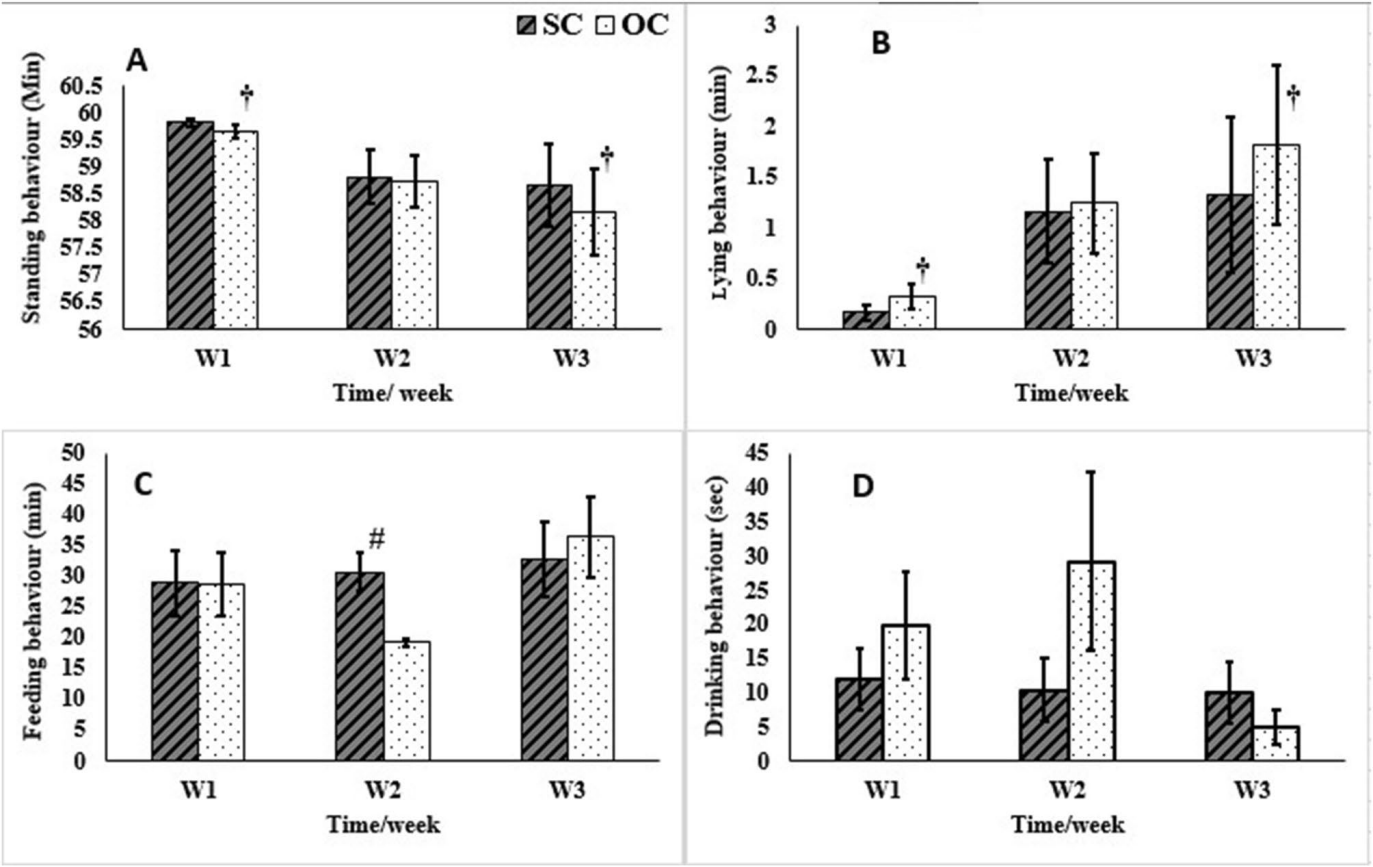
Standing (**A**), laying (**B**), feeding (**C**), and drinking (**D**) behavioral activities following subcapsular (SC) and open (OC) castration in donkeys during the 1st, 2nd, and 3rd weeks (W) post-castration. ^#^Indicates significant difference between different groups, *Indicate significant difference at different time intervals within the SC, ^†^Indicate significant difference at different time intervals within the OC.

During the 1st week (W1) post-castration, there were no significant changes (*P* > 0.05) in different behavioral activities between the SC and OC groups; except for the tail wagging activity that significantly increased (*P* ≤ 0.001) in the SC group compared to the OC group (Fig. 7C). The walk and feeding activities increased significantly (*P* = 0.021 and 0.000, respectively) in the SC group compared to the OC group during the 2nd week (W2) post-castration (Figs. 7A, 8C). There was a significant increase (*P* ≤ 0.001) of penis exposure and a significant decrease in testicles suckling and pelvic limb lifting (*P* = 0.01 and 0.03, respectively) in donkeys of the SC compared to the OC group during W2 and the 3rd week (W3) post-castration (Fig. 7D–F).

Donkeys of the SC group exhibited a significant increase (*P* ≤ 0.001) in the walk and exposing penis activities on W2 and W3 compared to W1 post-castration (Fig. 7A,D). However, there were no significant changes (*P* > 0.05) in the grooming, tail wagging, testicle suckling, pelvic limb lifting (Fig. 7B,C,E,F), standing, lying, feeding, or drinking activities (Fig. 8A–D) in donkeys of the SC group.

Donkeys of the OC group exhibited a significant increase (*P* ≤ 0.001) in the tail wagging, exposing penis, testicle suckling, pelvic limb lifting (Fig. 7C–F), and laying activities (Fig. 8B) on W3 compared to W1 and W2 post-castration. The standing activity significantly decreased (*P* = 0.03) on W3 compared to W1 post-castration (Fig. 8A) in the OC group. There were no significant changes (*P* > 0.05) regarding the walk, grooming (Fig. 7A,B), feeding, or drinking activities in donkeys subjected to the OC technique (Fig. 8C,D).

Regarding the pain score, there was a numerical decrease in donkeys of the SC group, contrary to a numerical increase in donkeys of the OC group over time. Donkeys of the SC group exhibited a significant decrease (*P* = 0.007) in the pain score on days 7 and 15 post-castration compared to the donkeys of the OC group. However, there were no significant changes (*P* > 0.05) on days 3 and 21 post-castration between the SC and OC groups (Table 2).

**Table 2:** Mean rank of pain score of donkeys subjected to subcapsular (SC) and open castration (OC) techniques.

Type of castration	Pain score/Days				P value
	3	7	14	21	
SC	6.50	3.83	3.83	4.83	*0.163*
OC	6.50	9.17	9.17	8.17	*0.498*
P value	*1.000*	*0.007*	*0.007*	*0.080*	

Differences were considered statistically significant for P < 0.05.

The pain score differences were determined using the Kruskal–Wallis nonparametric test due to lack of normality distribution.

## Discussion

This study described the subcapsular technique for primary closure castration in donkeys. Testicular tunics were closed after excision of the parenchyma bilaterally through a single paramedian scrotal incision. The cremaster muscle, ligament of the tail of the epididymis, and scrotal ligament were left intact in place. The procedure was successfully performed and tolerated in donkeys, with minimal incidence of postoperative complications, good cosmetic results, and less aftercare post-surgery.

The choice of an appropriate surgical approach of equine castration depends on many factors, including; the age of the animal, size of the testicles, presence of certain clinical circumstances (e.g., cryptorchidism, eventration, testicular neoplasms), and preference of the surgeon^2,3,13,26^. However, each technique has advantages and disadvantages. The standing castration is not recommended in donkeys as a whole due to their relatively short height and lack of good visualization of anatomical structures. Although the open technique is simple and allows a good crushing to the spermatic cord structures, vaginal tunics and scrotal wounds left open increase the risk of infection and eventration^9,27^. On contrary, even though the closed technique maintains no access to the abdominal cavity, it is not recommended for older animals that have heavily deposited scrotal fat and spermatic cord may not be adequately crushed^3^. The semi-closed technique provides adequate crushing of the spermatic cord and inguinal approach for non-descended testicles, but the incision of the parietal vaginal tunic increases the risk of postoperative infection. In addition, it is not recommended in cases of inguinal hernias, where the puncture of herniated intestine can occur^3,5^. Kummer et al.^28^ described a novel technique for primary castration via an inguinal approach with a low complication rate compared with other castration techniques. Laparoscopic castration is a less invasive procedure; however, such technique requires expensive equipment and an experienced surgeon^29^.

Here, The SC technique was associated with less postoperative complications than in the OC technique. Donkeys in the SC group did not experience a postoperative infection or excessive edema as in the OC group. This may be attributed to re-suturing of the testicular tunics, preserving of the cremaster muscle, tail of the epididymis, and scrotal ligament intact in place, and the primary closure of the scrotal incision. This was in accordance with previous reports of Barber^30^, Sedrish and Leonard^5^, and Saifzadeh et al.^8^. Moreover, the scrotal approach through a relatively short cutaneous incision as in the SC technique provided good cosmetic outcomes and reduced the risk of postoperative infection.

There was minimal bleeding following surgical excision of the testicular parenchyma, which was successfully controlled through the cauterization in donkeys of the SC group. Also, the closure of the albuginea and visceral vaginal tunics decreased the chance of hemorrhage occurrence post-castration. This might be advantageous rather than surgical intervention with the spermatic cord vasculature, which has been approached in all procedures of surgical castration in equines. The testicular artery and vein in donkeys are larger than in horses, which increase the chance of hemorrhage occurrence after surgery. Moreover, excessive pulling of the spermatic cord while placing the ligature (that can cause the vessels to retract inside) or inadequate ligature could be potential causes for post-castration hemorrhage during such procedures^3,11^.

Scrotal hemorrhage was recorded in one donkey of the OC group in this study. This may be attributed to the presence of significant blood supply to the scrotum of mature donkeys^3^. Wounds left open in the OC procedure increased the risk of infection and were the cause that donkeys of the OC group required some degree of aftercare than in the SC^2,5,27^. The SC procedure consumed a relatively longer time (43.33 ± 3.33 min) than in the OC one (9.33 ± 0.66 min). However, it was shorter than in other techniques as the modified open technique (60 min, range: 40–90 min)^9^. Tying the uppermost hind limb provided adequate exposure to the surgical area. Also, the lower testicle was first operated to ensure good visualization of the second one^3^. The intratesticular and intrafunicular infusion of lidocaine HCl 2% provides an antinociceptive effect even post-castration due to its long-lasting effect^31^. Hand-walking on the first five days post-castration is recommended to dissipate swelling and enhancing wound drainage^2,5^.

The similar pattern of testosterone depletion in the SC and OC groups from the end of the surgical operation after that demonstrates the equipotent effectiveness of both types of castration consistent with that reported in patients with prostate cancer^17,20^. This could be due to the removal of the testicular parenchyma, the main site of male sex hormones^32^.

Orchiectomy causes activation of hypothalamic–pituitary–adrenal axis leading to increased adrenocorticotrophic hormone and cortisol concentration^33^. The increase in cortisol level at the end of the surgery is concomitant with that observed in piglets undergoing castration^34^ and calves undergoing dehorning^35^. The type of surgical incision made could be an important factor in determining the strength of stressful stimuli^36^. Therefore, a single vertical paramedian scrotal incision is suggested to be a less invasive method compared to the castration technique that makes two incisions one over each testicle; due to the polymodal nociceptors in the skin.

Our finding does not necessarily indicate a stress response to the SC method, especially that the outcome was transient and rapidly disappeared along the next time intervals. As the castration reduces the testosterone levels and subsequently the aggressive behaviors, and therefore can help to reduce the levels of physiological stress indicators such as cortisol^37^. It is assumed that stress can cause both increased and decreased reaction of the hypothalamic–pituitary–adrenal axis at different time points during a stressful situation: hypothalamic–pituitary–adrenal activation and elevated cortisol levels at the beginning can be followed by a counter-regulatory response over time by feedback mechanisms^38^. Circadian secretion pattern^39^, confounding factors other than pain, and requirement of frequent sampling to capture periodic fluctuations^40^ are obstacles that stand in front of using of cortisol as a valid parameter for measuring the magnitude of stress and pain. Supporting this suggestion, the current study reported a reduction in pain score and return to normal behavioral patterns and disappearance of pain associated behavioral activities in the SC group earlier than in the OC group.

The obvious increase in serum glucose level on day 7 following the SC versus the baseline matched with the findings in castrated rats^41^. This may be attributable to the ability of castration to induce insulin resistance^42^ and gluconeogenesis and decrease muscle glucose uptake^41^.

Lactate is a biochemical measure of the metabolic changes related to painful situations. Catecholamines release in these conditions lead to augmented glycogenolysis and mobilization of muscular glycogen, and its consequent hepatic metabolism resulting in increase in lactate output^43^. This explained the marked increase in lactate level in the OC group compared to the SC group similarly to that presented in surgically castrated piglets^36^. However, the reduction in lactate level in the OC group on days 2, 15 and 30 post-castration compared to day 0 could be due to its utilization as a glucogenic precursor in gluconeogenesis pathway. Castration induces insulin resistance, which suppress glucose consumption in skeletal muscle increasing its lactate production. Hepatic lactate uptake is enhanced following castration contributing to increased hepatic gluconeogenic activity via utilization of lactate as a glucogenic precursor^44^.

Inhibition and down-regulation of NO synthase and up-regulation of asymmetric dimethylarginine, an arginine compound decrease NO bioavailability^45^, could be reasons underlying the reduction in NO level following the SC at last point in the studied time frame compared to day 0. The reduction in serum TC level in the OC group at the final time point compared to the baseline consistent with that observed in surgically castrated donkey^46^. This finding is possibly owing to down-regulation in the transcript level of hepatic 3-hydroxy-3-methylglutaryl-CoA reductase; a rate-limiting enzyme in cholesterol synthesis^47^. Castration enhances the cellular uptake of cholesterol and fatty acids and inhibits lipolysis causing depletion of TC from circulation. For example, gene expression of lipase G was up-regulated while that of phosphatidic acid phosphatase type 2B was down-regulated in the androgen-deprived pig model reflecting increased translocation of lipids from the extracellular space to the lipid storage sites and inhibition of lipolysis^48^. The increase in TG in the OC group on day 7 post-castration versus the baseline is matched with that found in rabbits^49^ presumably due to increased TG synthesis in liver through enhancing lipogenesis and free fatty acid uptake induced by hypogonadism^50^.

This study explored the behavioral changes presented by donkeys subjected to either the SC or OC techniques. Donkey pain scoring systems are often based in whole or in part on subjective assessment of postures or states that indicate decreased alertness, activity, or response^14,25,51–53^. Most of the significant behavioral changes were recorded during W2 and W3 post-castration. This may be attributed to the effect of the analgesic and anti-inflammatory effect of the post-operative treatments during W1.

Increased walking, feeding, and exposing penis, in addition to lack of the pelvic limb lifting or testicle suckling activities in donkeys of the SC group over time reflected rapid health status improvement and low incidence of pain-associated behaviors in the SC group. This was in accordance with the recorded low numerical pain score of the SC group. This may be attributed to the less-invasive SC technique that allows the approach of both testicles through a single short scrotal cutaneous incision with subsequent closure achieving healing by the primary intention type.

In contrast, the increase in the standing, testicular suckling, and lifting of the pelvic limb, as well as, the decrease in the lying and tail wagging activities of donkeys in the OC group may be due to pain associated with the bilateral opened scrotal wounds, other than the invasive severing of the spermatic cords bilaterally in the OC technique. This was in accordance with Ting et al.^54^ who reported that open surgical castration increased standing and reduced the lying postures.

Oliveira et al.^16^ reported that the pelvic limb lifting is the only specific behavioral indicator of pain after castration in donkeys, while, the tail, head, and ear movements are non-specific responses may be related to the presence of insects in a dirty stall. Also, Regan et al.^51^ reported that tail wagging could be an attempt to get some pain relieve from biting insects. The sustained pain and inflammation associated with the open castration might obscure the tail wagging activity of donkeys in response to fly biting, while increased the pelvic limb lifting, in attempts to relieve pain from the scrotum. In contrast, the limited inflammation in donkeys of the SC group accompanied with increased tail wagging and lack lifting of the pelvic limbs.

However, the described SC technique in this report may not be the appropriate for equine castration in case of cryptorchid testes, which can locate either in the inguinal canal or in the abdomin or in case of enlarged testicular tumors. Also, it was relatively time consuming and could be considered a complicated procedure by inexperienced practitioners. Moreover, it should be accomplished under complete aseptic conditions in medical institutions rather than in field conditions.

## Conclusion

The subcapsular castration was an efficient technique for castration with primary wound healing in donkeys with minimal postoperative complications and care. It can be an alternative to other castration techniques in equines.

## Supplementary Information

Supplementary Information 1.

Supplementary Information 2.

## Data Availability

From the corresponding author.
