# Supplementary material for: Evaluation of the subcapsular technique for primary closure castration in donkeys (Equus asinus)
Source: Sci Rep. 2021 Jul 7;11:14080. doi: 10.1038/s41598-021-93585-y (PMC8263736; doi:10.1038/s41598-021-93585-y)
Supplement: Supplementary file 1 — Supplementary Information 1. [file 41598_2021_93585_MOESM1_ESM.docx]

**S1: Serum concentrations of testosterone and stress parameters in the subcapsular castration and open castration groups**

| **Time interval**  **Parameter** | **C** | **E** | **12 Hrs.** | **2 Days** | **7 Days** | **15 Days** | **30 Days** |
| --- | --- | --- | --- | --- | --- | --- | --- |
| **Testosterone level (mg/ml)** |  |  |  |  |  |  |  |
| **SC** | 1.96 ± 0.62 | 0.16 ± 0.03^*^ | 0.02 ± 0.00^*^ | 0.02 ± 0.00^*^ | 0.03 ± 0.00^*^ | 0.03 ± 0.01^*^ | 0.05 ± 0.02^*^ |
| **OC** | 2.29 ± 0.97 | 0.41 ± 0.15^†^ | 0.05 ± 0.02^†^ | 0.09 ± 0.05^†^ | 0.02 ± 0.00^†^ | 0.04 ± 0.02^†^ | 0.04 ± 0.02^†^ |
| **Cortisol level (µg/dl)** |  |  |  |  |  |  |  |
| **SC** | 4.84 ± 0.23 | 9.24 ± 0.60^*^ | 4.50 ± 0.42 | 6.19 ± 0.86 | 6.80 ± 0.64 | 4.15 ± 1.41 | 3.11 ± 1.15 |
| **OC** | 4.43 ± 0.04 | 7.19 ± 0.79 | 8.41 ± 2.47 | 5.90 ± 3.25 | 5.33 ± 0.35 | 3.87 ± 0.47 | 1.63 ± 0.77 |
| **Glucose level (mg/dl)** |  |  |  |  |  |  |  |
| **SC** | 14.47 ± 5.37 | 40.67 ± 13.75 | 29.25 ± 3.09 | 39.20 ± 6.75 | 75.79 ± 1.63^*^ | 23.06 ± 5.46 | 27.04 ± 5.66 |
| **OC** | 32.08 ± 1.45 | 27.67 ± 10.17 | 15.30 ± 7.79 | 72.54 ± 16.87 | 46.75 ± 8.63 | 36.48 ± 3.46 | 35.22 ± 9.80 |
| **Lactate level (mg/dl)** |  |  |  |  |  |  |  |
| **SC** | 29.35 ± 9.83 | 31.46 ± 8.69 | 57.18 ± 3.80 | 21.67 ± 2.69 | 19.80 ± 2.86 | 60.03 ± 14.00 | 37.24 ± 7.39 |
| **OC** | 94.59 ± 1.06 | 61.94 ± 6.95^#^ | 87.89 ± 7.52^#^ | 37.89 ± 4.37^#†^ | 61.26 ± 6.38^#^ | 54.97 ± 8.90^†^ | 45.34 ± 8.63^†^ |

SC: subcapsular castration; OP: open castration

^#^ Significant difference between both types of castration at the same time point

^*^ Significant difference in SC in comparison with the baseline level

^†^ Significant difference in OC in comparison with the baseline level
