# Supplementary material for: Evaluation of the subcapsular technique for primary closure castration in donkeys (Equus asinus)
Source: Sci Rep. 2021 Jul 7;11:14080. doi: 10.1038/s41598-021-93585-y (PMC8263736; doi:10.1038/s41598-021-93585-y)
Supplement: Supplementary file 2 — Supplementary Information 2. [file 41598_2021_93585_MOESM2_ESM.docx]

**S2: Serum concentrations of nitric oxide and lipid profile in the subcapsular castration and open castration groups**

| **Time interval**  **Parameter** | **C** | **E** | **12 Hrs.** | **2 Days** | **7 Days** | **15 Days** | **30 Days** |
| --- | --- | --- | --- | --- | --- | --- | --- |
| **NO level (nmol/ml)** |  |  |  |  |  |  |  |
| **SC** | 22.05 ± 0.54 | 23.52 ± 1.79 | 23.52 ± 0.69 | 22.05 ± 0.18 | 22.05 ± 0.63 | 21.95 ± 1.82 | 15.12 ± 0.31^*^ |
| **OC** | 19.95 ± 0.76 | 23.31 ± 3.58 | 23.73 ± 1.24 | 22.68 ± 0.36 | 28.56 ± 3.09 | 16.70 ± 3.34 | 13.97 ± 0.84 |
| **TC level (mg/dl)** |  |  |  |  |  |  |  |
| **SC** | 67.33 ± 2.67 | 66.22 ± 9.69 | 70.67 ± 12.10 | 72.00 ± 4.68 | 72.00 ± 3.15 | 56.44 ± 11.80 | 46.00 ± 8.34 |
| **OC** | 80.67 ± 2.78 | 70.22 ± 10.07 | 64.22 ± 15.47 | 72.89 ± 3.74 | 93.11 ± 9.94 | 56.67 ± 8.47 | 38.67 ± 3.33^†^ |
| **HDL-C level (mg/dl)** |  |  |  |  |  |  |  |
| **SC** | 30.21 ± 4.75 | 33.06 ± 4.87 | 35.72 ± 2.87 | 39.52 ± 3.64 | 37.24 ± 1.00 | 42.56 ± 5.94 | 41.33 ± 2.80 |
| **OC** | 41.23 ± 2.74 | 29.83 ± 2.76 | 27.17 ± 7.22 | 36.67 ± 3.13 | 39.90 ± 2.96 | 64.79 ± 8.33 | 45.03 ± 6.36 |
| **TG level (mg/dl)** |  |  |  |  |  |  |  |
| **SC** | 11.77 ± 0.23 | 16.34 ± 4.93 | 10.33 ± 1.36 | 10.20 ± 1.81 | 19.74 ± 3.14 | 14.64 ± 2.42 | 15.16 ± 3.52 |
| **OC** | 27.71 ± 4.08 | 22.09 ± 5.66 | 13.53 ± 1.70 | 20.78 ± 1.57^#^ | 58.63 ± 1.92^#^^†^ | 11.63 ± 2.88 | 16.47 ± 3.81 |

SC: subcapsular castration; OP: open castration; NO: nitric oxide; TC: total cholesterol; HDL-C: high density lipoprotein-cholesterol; TG: triglycerides

^#^ Significant difference between both types of castration at the same time point

^*^ Significant difference in SC in comparison with the baseline level

^†^ Significant difference in OC in comparison with the baseline level
